# Experimental Models in Syrian Golden Hamster Replicate Human Acute Pancreatitis

**DOI:** 10.1038/srep28014

**Published:** 2016-06-15

**Authors:** Yunan Wang, Abudurexiti Kayoumu, Guotao Lu, Pengfei Xu, Xu Qiu, Liye Chen, Rong Qi, Shouxiong Huang, Weiqin Li, Yuhui Wang, George Liu

**Affiliations:** 1 Institute of Cardiovascular Science, Key laboratory of Molecular Cardiovascular Science of Ministry of Education, Health Science Center, Peking University, Beijing, 100191, China; 2 Department of General Surgery, Jinling Hospital, Medical School of Nanjing University, Nanjing, 210093, China; 3 University of Cincinnati College of Medicine, Cincinnati, OH, USA

## Abstract

The hamster has been shown to share a variety of metabolic similarities with humans. To replicate human acute pancreatitis with hamsters, we comparatively studied the efficacy of common methods, such as the peritoneal injections of caerulein, L-arginine, the retrograde infusion of sodium taurocholate, and another novel model with concomitant administration of ethanol and fatty acid. The severity of pancreatitis was evaluated by serum amylase activity, pathological scores, myeloperoxidase activity, and the expression of inflammation factors in pancreas. The results support that the severity of pathological injury is consistent with the pancreatitis induced in mice and rat using the same methods. Specifically, caerulein induced mild edematous pancreatitis accompanied by minimal lung injury, while L-arginine induced extremely severe pancreatic injury including necrosis and neutrophil infiltration. Infusion of Na-taurocholate into the pancreatic duct induced necrotizing pancreatitis in the head of pancreas and lighter inflammation in the distal region. The severity of acute pancreatitis induced by combination of ethanol and fatty acids was between the extent of caerulein and L-arginine induction, with obvious inflammatory cells infiltration. In view of the advantages in lipid metabolism features, hamster models are ideally suited for the studies of pancreatitis associated with altered metabolism in humans.

Like mice and rats, Syrian golden hamsters are small rodent experimental animals widely used in medical study. They display many features that resemble humans in physiology, such as diet reactivity, metabolism, and infection of pathogenic microorganisms^1^,^2^,^3^. Recently, the application of genetic manipulation of embryonic cells in hamster made a success of generating transgenic or knockout hamster models^4^,^5^. Because of these combined features animal models for human metabolic diseases can be promisingly built on hamster in many cases instead of mice or rats in the future. Perhaps taking the advantage of phylogenetic similarities of hamsters to humans in pancreas features^6^, there were a few reports using hamster as the animal model in the study of pancreatitis^7^,^8^. Therefore, in hamster, the present study tested 4 reproducible models of acute pancreatitis generally used in mice and rat before, including peritoneal injections of caerulein or L-arginine, retrograde infusion of sodium taurocholate, and concomitant ip injection of palmitoleite and ethanol. These models could lead to a promising new approach using genetically modified hamster models in the future for studies on acute pancreatitis.

## Results

### Hamster anatomical characteristics of the pancreas

**Figure 1:**
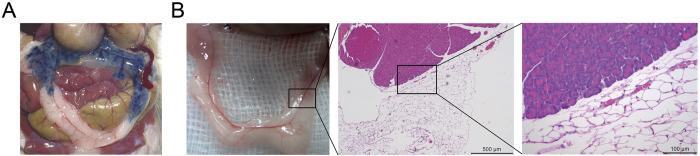
Anatomical characteristics of hamster pancreas. Adjacent to duodenum, part of pancreas bypasses the stomach, extends to the spleen, and further circulates along the right side of abdomen. They are joined in the end by the adipose tissue. (**A**) The infusion of trypan blue through pancreatic duct showed the boundary of pancreas tissue. (**B**) The pancreatic tissue and the adipose tissue of hamster showed different colors. The pancreatic tissue was stained with HE method and the contiguous area was shown with magnification of 50X and 200X.

We found hamsters have different anatomy in pancreas from mice and rats, as shown in Fig. 1. The characteristic feature of hamster pancreas is the junction of adipose tissue to the tail of pancreas tissue like human. The infusion of trypan blue through pancreatic duct showed the boundary of pancreas tissue (Fig. 1A). The hematoxylin & eosin (HE) staining also demonstrated the transition of pancreas tissue to adipose tissue to the best advantage. Therefore, hamster may provide some benefits to the study of pancreatitis associated with adipose factors.

### Increased plasma amylase level in caerulein, L-arginine, Na-taurocholate, and ethanol + POA-induced models by different tendency in timing

**Figure 2:**
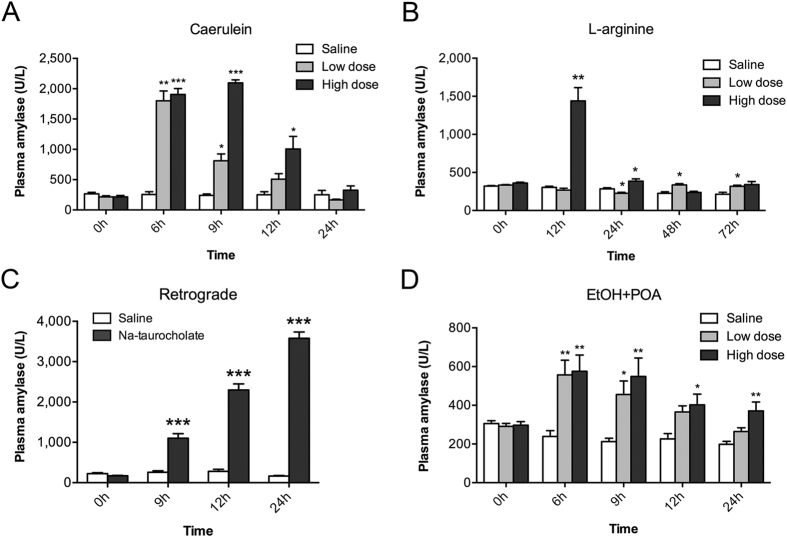
Plasma amylase levels of hamsters after induction of acute pancreatitis by (**A**) caerulein, (**B**) L-arginine, (**C**) Na-taurocholate and (**D**) Ethanol (EtOH) + POA. *p < 0.05, **p < 0.01, and ***p < 0.001 were obtained in comparison to the saline group for n = 8 in each group.

Elevated plasma amylase is an important biomarker for the damage of pancreatic acinar cell. Four induction methods all resulted in significantly increased plasma amylase activity, but the peak of the activity appeared in different hours after induction. Acute pancreatitis usually was induced by 4 injections of caerulein in rats^9^, and by 7 injections in mice^10^ as reported previously. Like mice^9^ 9 hours after the first injection of caerulein by 7 times, and like rat^10^ 6 hours after the first injection of caerulein by 4 times, the amylase activity of the hamsters reached the maximum in plasma (Fig. 2A). The peak of amylase activity in Na-taurocholate-induced hamster appeared at the 24 hours after infusion of the pancreatic duct (Fig. 2C), which is also consistent with the pick of activity in mice^11^ and rats^12^. Different from the reports on the L-arginine-induced pancreatitis in mice^13^ or rats^14^, the plasma amylase level in L-arginine-induced pancreatitis in hamsters reached peak at 12 hours after first injection of high dose (3 g/kg), and there was no significant increase of plasma amylase level in low dose (Fig. 2B). Because of the inconsistence in the two reports^15^,^16^ of Huang W *et al*., we performed two intraperitoneal injections of 1.35 g/kg ethanol (EtOH) and 2 mg/kg palmitoleic acid (POA) or 150 mg/kg POA according to the two reports. We found the similar pattern of increased serum amylase (peak at 6 hr) after induction of ethanol with two doses of POA (Fig. 2D). However, the high dose of POA could induced higher serum amylase level at 24 hours. These results suggest that hamsters exhibit the similar changes in serum amylase like other model animals.

### Exhibition of classic pathological changes in pancreas in the four acute pancreatitis models

**Figure 3:**
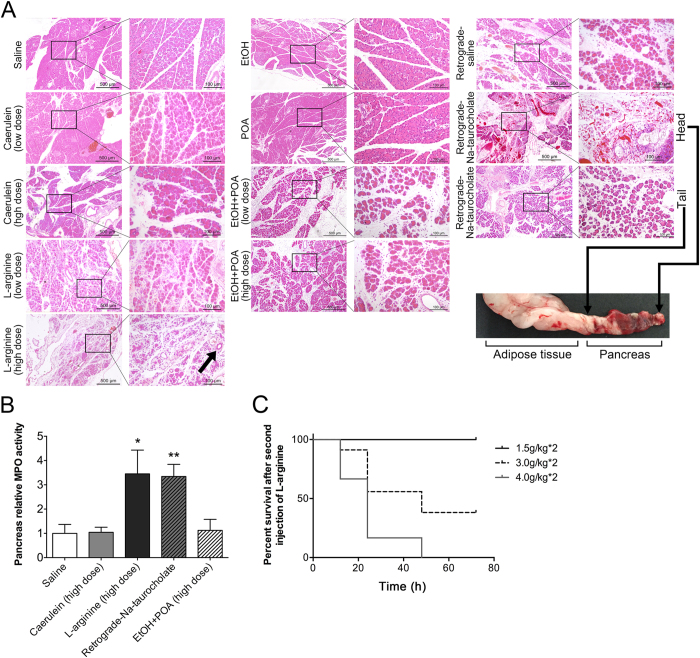
Representative pathological changes in pancreatitis induced by caerulein, L-arginine, Na-taurocholate and ethanol (EtOH) + POA respectively. (**A**) HE stained sections of pancreas in magnification 50X or 200X. The arrow on left panel shows vacuolization in pancreas tissue from L-arginine-induced model, and arrows on right panel indicate the head and the tail of pancreas from Na-taurocholate-induced model, respectively. (**B**) Relative MPO activity of pancreatic tissue in 4 different pancreatitis models. *p < 0.05 and **p < 0.01 were obtained in comparison to the saline group for n = 8 in each group. (**C**) Survival curve after L-arginine injection.

According to the different timing of the amylase changes, the pancreas tissues were collected at different time points from these 4 pancreatitis models, specifically 24 hours in caerulein, 72 hours in L-arginine, 24 hours in Na-taurocholate, and 24 hours in ethanol + POA-induced. The pathology of pancreas was examined with anatomical observation and HE staining. After induction of acute pancreatitis, pancreas tissue exhibited edema, necrosis, inflammatory infiltration and acinar hemorrhage, supporting a conventional pathological changes of pancreatitis in caerulein, L-arginine, Na-taurocholate and ethanol + POA-induced models (Fig. 3A). Relatively, caerulein induced a minor acute pancreatitis mainly with edema and neutrophil infiltration; and L-arginine induced a severe acute pancreatitis mainly with acinar necrosis, heavy inflammatory cell infiltration and a high mortality rate (Fig. 3C). Na-taurocholate also induced a severe acute pancreatitis in the anterior of pancreas but slight morphologic changes, such as necrosis and inflammatory infiltration, in the posterior of pancreas (Fig. 3A). Ethanol + POA-induced acute pancreatitis exhibited the severity between caerulein and L-arginine induction with obvious edema, a large number of neutrophil infiltration, and acinar necrosis (Fig. 3A). There is no difference between 2 mg/kg and 150 mg/kg POA in ethanol + POA-induced models. Ethanol or POA alone did not induced the pathological changes in pancreas (Fig. 3A).

The pancreatic tissue of hamster is larger than that of mouse, so that there is more quantity of pancreatic tissue sample for the measurement of MPO activity. Hamsters induced by L-arginine and Na-taurocholate had significantly increased pancreatic MPO activity respectively (Fig. 3B), but the changes in MPO activity did not reveal the neutrophil infiltration in caerulein- and ethanol + POA-induced pancreatitis. We considered that it may be limited by the sensitivity of our measurement.

Based on a standard for evaluation of acute pancreatitis as previously described^17^ (Table 1), semi quantitative morphological scores showed the significant pancreatic injury in all induced pancreatitis models (Table 2), in which L-arginine and Na-taurocholate-induced models display much more severe pathological characteristics than caerulein-induced pancreatitis, and the severity of ethanol + POA-induced model was in between.

### Different degrees of lung injury displayed in the four models

Among multiple organ damage in acute pancreatitis, lung injury is the most common one. In a preliminary experiment, we did not find an obvious injury in heart, liver, spleen and kidney in 4 pancreatitis models. However, interstitial tissue thickness, neutrophil infiltration, alveolar septum breakage, and pulmonary alveoli hemorrhage were found in lung pathological sections, which is similar to what has been reported in human or other rodent models.

**Figure 4:**
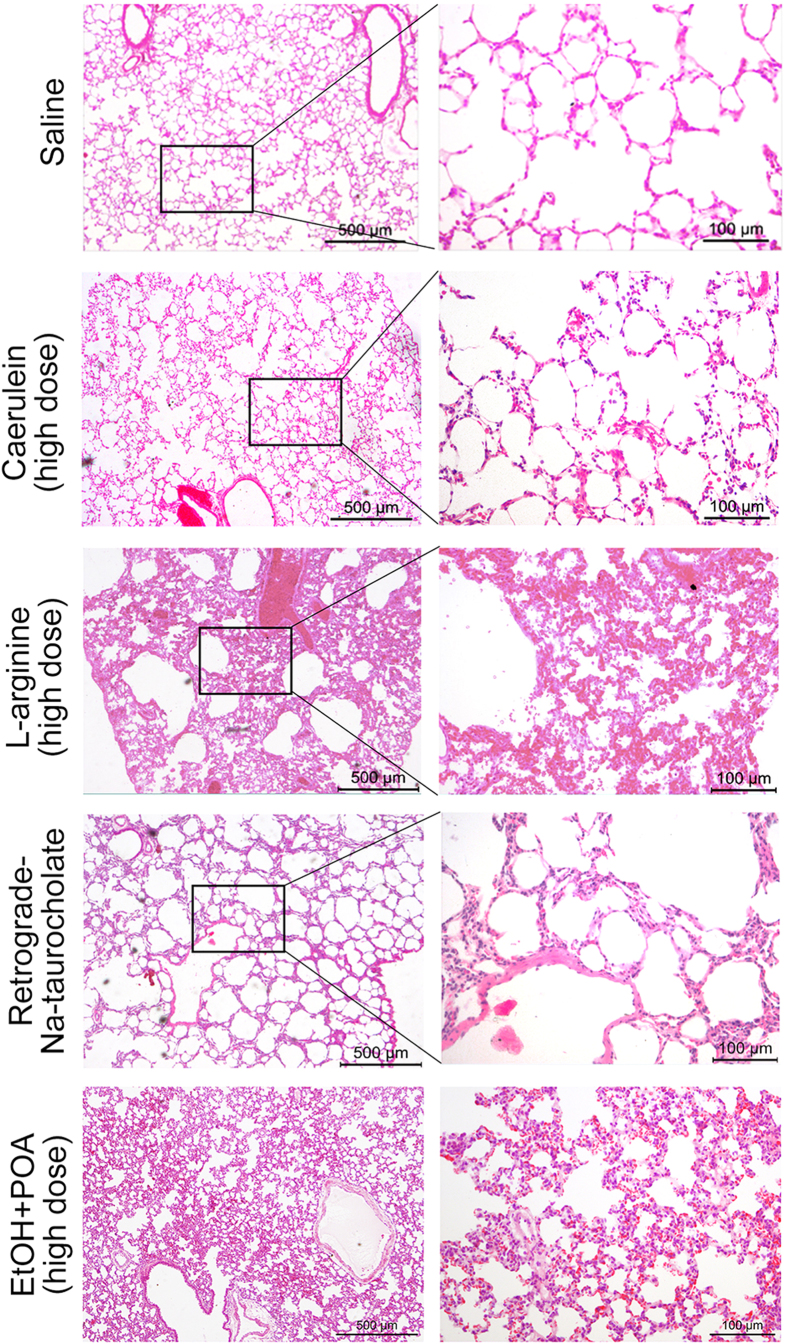
HE stained sections of hamster lung tissue showed pathological changes in caerulein, L-arginine, Na-taurocholate and ethanol (EtOH) + POA induced models respectively. Lung tissues were injured obviously in L-arginine and Na-taurocholate induced pancreatitic models.

In caerulein model, slight pulmonary alveoli injury and inflammatory cell invasion without elevated thickness of the alveolar septum or occurrence of hemorrhage were observed (Fig. 4). Accompanying severe acute pancreatic injury, administration of L-arginine caused severe lung injury (Fig. 4), including even more serious alveolar septum thickness, neutrophil infiltration, hemorrhage, and alveolar septum break. As another severe acute pancreatitis model, lung injury of Na-taurocholate-induced model was not as severe as L-arginine-induced. Their pathological changes mainly showed alveolar septum thickness and some hemorrhagic lesions (Fig. 4). Lung injury in ethanol + POA-induced model was similar to Na-taurocholate induced model characterized by alveolar septum thickness and hemorrhage (Fig. 4).

### Significant increase of pancreatic inflammatory factor IL-6 and TNF-α in acute pancreatitic hamster models

**Figure 5:**
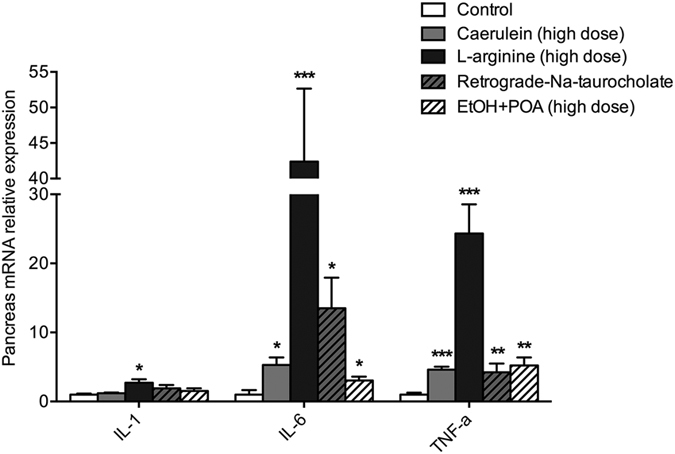
The expression of inflammatory factors in pancreas of caerulein, L-arginine, Na-taurocholate and ethanol (EtOH) + POA induced models. *p < 0.05, **p < 0.01, and ***p < 0.001 were obtained in comparison to the saline group, n = 8 in each group.

Generally, the mRNA expression of inflammatory factor in the pancreas can reflect the severity of pancreatitis. We examined IL-1,IL-6 and TNF-α expression levels, and the results showed a significantly enhanced expression level of IL-6 and TNF-α in all of the induced pancreatitic models. The expressions of IL-6 and TNF-α in L-arginine-induced group which had the highest morphological scores increased over 20 times to those of control group (Fig. 5). The level of IL-6 expression of Na-taurocholate group was the second highest, while its expression of TNF-α was about the same as caerulein-induced model. The markedly enhanced IL-6 and TNF-α expression provide additional cytokine biomarkers for evaluating the inflammatory regulation in pancreatitic models. However, the expression of IL-1 is not highly enhanced in all 4 models, suggesting that IL-1 is probably not an appropriate biomarker for assessment of the severity of pancreatitis in hamster’s models.

## Discussion

Acute pancreatitis is a severe inflammatory disease of the pancreas that can lead to multiple organ failure and result in significant mortality^18^. In this study, we successfully established 4 acute pancreatitis models in Syrian golden hamsters utilizing conventional methods that have been used in mice and rats previously. The hamster models not only present significant changes in plasma amylase level, pancreatic tissue pathological injury (edema, inflammatory cell infiltration, necrosis, hemorrhage, and vacuolar degeneration), pancreatic MPO activities, pancreatic inflammatory mRNA expression level and lung injury, but also simulate the anatomical features of human pancreas. In addition, our data showed that caerulein-induced model mimics the mild edematous pancreatitis and L-arginine-induced model stimulates perhaps the severer necrotic pancreatitis. While the Na-taurocholate-induced model resembles the gallstone-induced pancreatitis, the ethanol/POA induced model could represent the acute alcoholic pancreatitis. Therefore, these 4 hamster models can be applied for different purposes of investigation of acute pancreatitis.

Caerulein-induced hamster model was relatively stable and dose-dependent. High dose (50 μg/kg × 7 injections) of caerulein induced pancreatitis exhibiting typical edema and necrosis, and significantly increased plasma amylase level, morphological scores of pancreatic tissue and the expression of inflammatory factors in pancreas. The peak of plasma amylase activity emerged at the 9 hours after the first injection to the hamsters, suggesting hamster may be more susceptible to caerulein than mice or rats, whose amylase activity peaked at 12 hours after the first injection in most reports.

L-arginine-induced pancreatitis models in mice and rats are not commonly used due to the high mortality, small efficient dose window, instability as well as large individual variations. Most of the earlier methodological studies of the models focused on adjustment of drug concentration, doses and injection times^19^. In our experiment in hamster, there were not large individual variations. We observed severer necrosis in pancreatic tissue by injection of L-arginine for 2 times at 1.5 g/kg and wide-spreading hemorrhage at 3.0 g/kg. Over 90% of the acinar cells were necrotic and plasma amylase level, pancreatic MPO activity and the expression of inflammatory factors were all significantly increased by high dose treatment. We therefore consider that hamster is more suitable for pancreatitis model with L-arginine induction. It is noteworthy that the mortality rate of low dose L-arginine-induced group was zero in hamster, unlike mice and rats as described in previous reports^13^,^14^. We also found a special pathological characteristic of vacuolization in this model (Fig. 3A).

Biliary tract stone entering terminal biliopancreatic duct is considered to be the most frequent triggering event in acute pancreatitis in humans^20^. Na-taurocholate-induced model resembles the gallstone-induced pancreatitis in human. Our results showed that a large area of hemorrhage and necrosis occurred mainly in the anterior but not the posterior of hamster pancreas, which is similar to the pathological characters of mouse^11^ and rat^12^ models. However, the mortality of the model in the hamster is very low unlike mice^11^, and hamsters have no ascitic fluid accumulation unlike rat^12^ either.

The generation of FAEEs in pancreas has been demonstrated as the pathogenesis of alcoholic acute pancreatitis in mice^21^,^22^. Ethanol/POA combined intraperitoneal administration is considered as a novel FAEE-induced acute pancreatitis model. We applied this method to hamsters and the results obtained also showed similar characteristics in pathological changes as in mice. As reported previously^3^, hamsters are special rodents that respond strongly to high fat diet so that hamsters had been used as useful animal model for studies on the diseases associated with lipid metabolism. Acute pancreatitis is also well known to be associated with altered lipid metabolism conditions^23^, such as severe hypertriglyceridemia^8^,^15^, obesity^24^, and even diabetes^25^, all of which are believed to sensitize the pancreas to other stimuli of pathogenic factors. From this point, we consider that ethanol + POA-induced model or other models created by the methods associated with free fatty acids may be preferable to the studies of the relationship between acute pancreatitis and disturbed lipid metabolism.

Our previous study^8^ had proven that the local high concentration of free fatty acids in hypertriglyceridemic mice resulted in increased susceptibility to acute pancreatitis. We also had found enhanced susceptibility to acute pancreatitis in hypertriglyceridemic Syrian golden hamsters^26^. Therefore, we believe that with the generation of genetically modified hamster models in lipid metabolism, this species will be widely used for investigation in acute pancreatitis.

## Materials and methods

### Animals

Hamsters weighing approximately 150 g were purchased from Vital River Laboratories (Beijing, China). Animals were maintained on a 12-hour light/12-hour dark cycle at 24 °C with standard laboratory chow and water ad libitum. The Principles of Laboratory Animal Care (NIH publication no. 85Y23, revised 1996) was monitored during the study. All experiments were performed according to the protocol approved by the Animal Care Committee, Peking University Health Science Center (LA2015012). The hamsters were fasted overnight before induction of acute pancreatitis and randomly divided into an experimental group and control group with a number equal to 10 each.

### Induction of acute pancreatitis

Sterile solution of caerulein (Sigma Aldrich Chemie GmbH, Steinheim, Germany) was prepared in saline with 0.01% BSA at the concentration of 0.005%. The sterile solution of caerulein was injected intraperitoneal to induce acute pancreatitis by 7 or 4 injections interval hourly at 50 μg/kg. The hamsters injected with saline alone served as pancreatitis-free controls.

Sterile solution of L-arginine hydrochloride (Sigma Aldrich Chemie GmbH, Steinheim, Germany) was prepared in normal saline at the concentration of 8% and pH 7. Intraperitoneal injection of the sterile arginine solution at a dose of 1.5-6 g/kg twice hourly served as acute pancreatitis group, and the pancreatitis-free controls were injected with saline alone.

According to Laukkarinen’s protocol^11^, we used Na-taurocholate (2%, 40 mg/kg) to induce pancreatitis in hamsters. Sterile solution of Na-taurocholate (Sigma- Aldrich, St Louis, Missouri, USA) was prepared in normal saline at the concentration of 2%. Hamsters were anesthetized with ketamine (100 mg/kg, Fort Dodge Animal Health, Fort Dodge, Iowa, USA). A midline incision was made to expose the duodenum with attached pancreatic head, and then holding in place with ligatures flipped the duodenum’s distal side. Parallel to the papilla of water, a small puncture was made with a 23G needle. A transfusion needle (30G) connected to micro-infusion pump was introduced 2 mm into the pancreatic duct. To prevent hepatic reflux, the common hepatic duct was clamped with a small bulldog clamp. 300 μl of saline or Na-taurocholate solution was infused into the pancreatic duct at a rate of 30 μl/min. Before suturing the abdominal wall, the bile duct clip was removed and the duodenal puncture wound was closed.

Based on Huang’s reports^15^,^16^, palmitoleic acid (POA, Sigma-Aldrich, St. Louis, MO, USA) and ethanol were used to establish ethanol + POA-induced pancreatitis. Briefly, hamsters received 2 intraperitoneal injections of ethanol (1.35 g/kg) and POA (2 mg/kg or 150 mg/kg, diluted in DMSO) at 1 h intervals. 500 μL normal saline was injected immediately prior to ethanol/POA injections in case that ethanol or POA may cause local damage to the injection site. Control hamsters received either DMSO, ethanol (1.35 g/kg) or POA (2 mg/kg or 150 mg/kg).

### Measurement of plasma amylase

Blood samples were collected just before the induction of pancreatitis and 6, 9, 12 and 24 hours after the first injection of caerulein, ethanol, or infusion of Na-taurocholate. For L-arginine induction, blood was collected at 12, 24, 48 and 72 hours after injection of L-arginine. The blood was centrifuged at 40,000 rpm for 10 minutes at 4 °C to separate plasma. Amylase activity was measured with 5-ethylidene-G7PNP as a substrate by a commercial kit (Beijing Zhongshengbeikong Biochemistry Company).

### Histological examination of the pancreas and lung injury

Pancreatic tissues and lung were collected after 24 hours or 72 hours after induction of pancreatitis and the tissues were fixed in 4% paraformaldehyde in PBS (pH 7.4). Paraffin embedded tissues from each hamster were sectioned at 3 μm and then performed with H&E staining. Two investigators who were blind to the experimental treatment scored the degree of pancreatic injury by light microscopy for evaluating the severity of edema, inflammation, necrosis, hemorrhage, and vacuolization according to the standard described previously^17^ as the Table 1.

### Measurement of tissue MPO activity

Briefly, the tissues were thawed and homogenized in 50 mM phosphate buffer (10 ml/mg, pH 7.4) containing protease inhibitors and the centrifuged pellet was washed twice. The pellet was resuspended in 100 mM phosphate buffer (pH 5.4) containing 0.5% hexadecyl-methyl-ammonium bromide and 10 mM EDTA and was disrupted three times by sonication and freeze thaw cycles. The supernatant obtained after centrifuging was used for the MPO assay with 3,3’,5,5’-tetramethylbenzidine as the substrate. The protein concentration in the supernatant was measured by the pyrogallol red molybdate protein dye binding assay (Thermo, Rockford, USA). Relativity MPO activity was expressed as absorbance corrected by the amount of protein.

### Measurement of inflammatory factors expression

Total RNA was isolated from pancreas tissue using Trizol reagent according to the manufacturer’s recommendations. RNA was reverse-transcribed, Trizol reagent (Invitrogen, Carlsbad, CA), and the first strand cDNA was generated with reverse transcriptase (Transgene, Beijing, China). Quantitative real-time PCR was performed with SybGreen (Invitrogen, Carlsbad, CA) to estimate the expression of inflammatory factors by (Agilent, Santa Clara, CA). 18S was used as the endogenous control gene for normalization. The primer sequences used were as follows:

IL-1: forward primer AGTCATTGTGGCTGTGGAGA, reverse primer TGTTGTTCATCTCGGAGCCT;

IL-6: forward primer CAACCCTGGCTGTATGGACA, reverse primer GTGCTCTGAATGACTCTGGCT;

TNF-α: forward primer CGGGCTGTACCTGGTTTACTC, reverse primer GGGCTCTTGATGGCGGAC;

18S: forward primer AGTCATTGTGGCTGTGGAGA, reverse primer TGTTGTTCATCTCGGAGCCT.

Fluorescent data obtained from real-time PCR was processed by comparative Ct method data analysis to obtain the relative quantity of target mRNA.

### Statistical Analysis

All data were presented as mean ± SD obtained from three or more independent experiments. Statistical comparisons among groups were performed by one-way ANOVA analysis. P < 0.05 was considered to indicate significant differences.

## Additional Information

**How to cite this article**: Wang, Y. *et al*. Experimental Models in Syrian Golden Hamster Replicate Human Acute Pancreatitis. *Sci. Rep.* **6**, 28014; doi: 10.1038/srep28014 (2016).

## Figures and Tables

**Table 1:** Morphological scoring of hamster pancreatitis.

Edema	Acinar necrosis
0 = absent	0 = absent
0.5 = focal expansion of interlobular septae	0.5 = focal occurrence of 1–4 necrotic cells/HPF
1 = diffuse expansion of interlobular septae	1 = diffuse occurrence of 1–4 necrotic cells/HPF
1.5 = same as1 + focal expansion of interlobular septae	1.5 = same as 1 + focal occurrence of 5–10necrotic cells/HPF
2 = same as 1 + diffuse expansion of interlobular septae	2 = diffuse occurrence of 5–10 necrotic cells/HPF
2.5 = same as2 + focal expansion of interacinarseptae	2.5 = same as 2 + focal occurrence of 11–16 necrotic cells/HPF
3 = same as 2 + diffuse expansion of interacinarseptae	3 = diffuse occurrence of 11–16 necrotic cell~HPF or foci of confluent necrosis
3.5 = same as 3 + focal expansion of intercellular septae	3.5 = same as 3 + focal occurrence of >16 necrotic cells/HPF
4 = same as3 + diffuse expansion of intercellular septae	4 =>16 necrotic cells/HPF (extensive confluent necrosis)
**Inflammation**	**Intrapancreatic hemorrhage**
0 = 0–1 intralobular or perivascular leucocytes/HPF	0 = absent
0.5 = 2–5 intralobular or perivascular leucocytes/HPF	2 = Focal occurrence in HPF
1 = 6–10 intralobular or perivascular leucocytes/HPF	4 = Diffuse occurrence
1.5 = 11–15 intralobular or perivascular leucocytes/HPF	
2 = 16–20 intralobular or perivascular leucocytes/HPF	
2.5 = 21–25 intralobular or perivascular leucocytes/HPF	
3 = 26–30 intralobular or perivascular leucocytes/HPF	
3.5 = more than 30 leucocytes/HPF or focal microabscesses	
4 = more than 35 leucocytes/HPF or confluent microabscesses	

**Table 2:** Results of morphological scoring in hamsters.

	Edema	Inflammation	Necrosis	Hemorrhage	Total
Saline	0.13 ± 0.35	0.00 ± 0.00	0.25 ± 0.46	0.00 ± 0.00	0.38 ± 0.52
Caerulein (low)	1.19 ± 0.26*	0.88 ± 0.64	0.38 ± 0.52	0.00 ± 0.00	2.44 ± 1.27
Caerulein (high)	3.00 ± 0.53*	1.56 ± 0.56*	1.75 ± 0.71*	0.00 ± 0.00	6.31 ± 1.31*
L-arginine (low)	2.56 ± 0.42*	2.81 ± 0.88*	3.13 ± 0.79*	2.75 ± 1.83	11.25 ± 3.25*
L-arginine (high)	4.31 ± 0.65*	4.00 ± 0.00*	4.00 ± 0.00*	4.00 ± 0.00*	16.31 ± 0.65*
Retrograde-saline	0.94 ± 1.08	0.75 ± 0.53	0.50 ± 0.53	0.50 ± 0.93	2.69 ± 1.91
Retrograde-Na-taurocholate	3.63 ± 0.52*^#^	3.25 ± 0.80*^#^	3.75 ± 0.53*^#^	3.75 ± 0.71*^#^	14.38 ± 1.43*^#^
EtOH	0.50 ± 0.32	0.5 ± 0.63	0.25 ± 0.27	0.00 ± 0.00	1.25 ± 0.88
POA	0.17 ± 0.26	0.00 ± 0.00	0.17 ± 0.26	0.00 ± 0.00	0.33 ± 0.41
EtOH + POA (low)	2.25 ± 0.69*^§¶^	2.25 ± 1.13*^§¶^	1.75 ± 0.82*^§¶^	0.33 ± 0.52	6.58 ± 2.29*^§¶^
EtOH + POA (high)	2.42 ± 0.66*^§¶^	2.67 ± 1.33*^§¶^	1.58 ± 0.74*^§¶^	0.17 ± 0.41	6.83 ± 2.79*^§¶^

Values are mean ± SD of 8 mice, statistical analysis was performed using the Mann−Whitney test, *p < 0.05 compared to Saline group, ^#^p < 0.05 compared to Retrograde-saline group, ^§^p < 0.05 compared to ethanol (EtOH) group, ^¶^p < 0.05 compared to POA group.
