# Corrigendum: Experimental Models in Syrian Golden Hamster Replicate Human Acute Pancreatitis

**DOI:** 10.1038/srep29645

**Published:** 2016-07-20

**Authors:** Yunan Wang, Abudurexiti Kayoumu, Guotao Lu, Pengfei Xu, Xu Qiu, Liye Chen, Rong Qi, Shouxiong Huang, Weiqin Li, Yuhui Wang, George Liu

Scientific Reports 6: Article number: 2801410.1038/srep28014; published online: 06152016; updated: 07202016

This Article contains typographical errors in the Discussion section.

“Our previous study^8^ had proven that the local high concentration of free fatty acids in hypertriglyceridemic mice resulted in increased susceptibility to acute pancreatitis. We also had found enhanced susceptibility to acute pancreatitis in hypertriglyceridemic Syrian golden hamsters^26^.”

should read:

“Our previous study^26^ had proven that the local high concentration of free fatty acids in hypertriglyceridemic mice resulted in increased susceptibility to acute pancreatitis. We also had found enhanced susceptibility to acute pancreatitis in hypertriglyceridemic Syrian golden hamsters^8^.”

